# Resilience of Freshwater Communities of Small Microbial Eukaryotes Undergoing Severe Drought Events

**DOI:** 10.3389/fmicb.2016.00812

**Published:** 2016-05-30

**Authors:** Marianne Simon, Purificación López-García, Philippe Deschamps, Gwendal Restoux, Paola Bertolino, David Moreira, Ludwig Jardillier

**Affiliations:** ^1^Centre National de la Recherche Scientifique, Unite d’Ecologie Systématique Evolution, Université Paris-Sud, AgroParisTech, Université Paris-SaclayOrsay, France; ^2^Génétique Animale et Biologie Intégrative, INRA, AgroParisTechParis, France

**Keywords:** protist, resilience, dormancy, plankton, 18S rRNA, temporal dynamics

## Abstract

Small and shallow aquatic ecosystems such as ponds and streams constitute a significant proportion of continental surface waters, especially in temperate zones. In comparison with bigger lakes and rivers, they harbor higher biodiversity but they also exhibit reduced buffering capacity face to environmental shifts, such that climate global change can affect them in a more drastic way. For instance, many temperate areas are predicted to undergo droughts with increasing frequency in the near future, which may lead to the temporal desiccation of streams and ponds. In this work, we monitored temporal dynamics of planktonic communities of microbial eukaryotes (cell size range: 0.2–5 μm) in one brook and one pond that experienced recurrent droughts from 1 to 5 consecutive months during a temporal survey carried out monthly for 2 years based on high-throughput 18S rDNA metabarcoding. During drought-induced desiccation events, protist communities present in the remaining dry sediment, though highly diverse, differed radically from their planktonic counterparts. However, after water refill, the aquatic protist assemblages recovered their original structure within a month. This rapid recovery indicates that these eukaryotic communities are resilient to droughts, most likely via the entrance in dormancy. This property is essential for the long-term survival and functional stability of small freshwater ecosystems.

## Introduction

As consequence of the global climate change observed since the late 19th century, strong meteorological episodes such as droughts are expected to rise in frequency and strength in the near future (IPCC, 2007), affecting terrestrial and aquatic ecosystems. If the effects of global warming are relatively well-documented for marine (e.g., Moran et al., 2010) and lacustrine (Perkins et al., 2010) environments, small freshwater ecosystems are often dismissed even though they will experience droughts more severely. Moreover, most of the studies carried out in this type of aquatic ecosystems have focused so far on particular taxa or phylogenetic groups, while investigations at the community level been less developed (Woodward et al., 2010).

Freshwater systems encompass isolated and fragmented habitats and, despite they represent only 0.8% of the Earth surface, harbor about 6% of all described species (Dudgeon et al., 2006). In particular, small freshwater ecosystems (averaging 100–1000 m^2^) are widespread, and numerically very important (3.2 × 10^9^ estimated water bodies), covering around 0.8 billion km^2^ altogether (Downing, 2010). They likely play non-negligible roles in biogeochemical cycles, especially in the carbon cycle, as they may sequester more organic carbon and produce higher amounts of CO_2_ than larger lakes (Downing, 2010). In addition, both small lotic and lentic ecosystems arguably are among the most fragmented habitats on Earth. This type of systems are highly variable in terms of water level, often having relatively short lifespan (Downing, 2010). Thus, evaporation, percolation and a higher water need by the surrounding plants may lead to complete desiccation during drought periods. Characterized by a multiplicity of physico-chemical parameter assortments and strongly influenced by seasonal changes in environmental conditions, small freshwater systems host a high biodiversity that, in what relates to its microbial component, is barely beginning to be explored (Downing, 2010; Simon et al., 2015a,b). However, how this biodiversity responds face to extreme environmental challenges, such as increasingly frequent droughts, remains virtually unknown.

Together with prokaryotes, microbial eukaryotes (protists) are key players in aquatic ecosystem functioning, being largely involved in carbon fixation (Li, 1994; Jardillier et al., 2010), nutrient cycling (Caron, 1994; Falkowski et al., 2008), toxin production (Scholin et al., 2000; Edvardsen and Imai, 2006) and control of prokaryotic and larger eukaryotic community members via predation and parasitism (Chambouvet et al., 2008; Worden et al., 2015). Studies on freshwater microbial ecology using state-of-the-art molecular methods based on 18S rRNA gene sequencing have mostly focused on large water bodies (permanent lakes) and, although a bias toward the bacterial component of microbial communities exists, microbial eukaryotes are increasingly explored (Lefranc et al., 2005; Richards et al., 2005; Zinger et al., 2012). Even if knowledge on small continental water bodies is more scarce, molecular surveys indicate that this type of systems sustain a high protist diversity (Šlapeta et al., 2005; Simon et al., 2015a) that undergoes seasonal dynamics (Lara et al., 2011; Simon et al., 2015b), consistent with large and rapid variations in their environmental conditions (Bamforth, 1958; Lara et al., 2011). Previous studies in freshwater seasonal variation (Lara et al., 2011; Simon et al., 2015b) suggest that members of the eukaryotic community may enter some way of dormancy (e.g., cyst or spore production, metabolic slowdown) and constitute a “seed bank” that participates to plankton community resilience over time. Dormancy is a distinctive property of prokaryotes, but also of many microbial eukaryotes, that allows them facing harsh environmental conditions and can contribute to maintain disturbed ecosystem functioning stable over time (Lennon and Jones, 2011). However, investigations on the impact of severe environmental challenges, such desiccation events during droughts, on freshwater microbial communities are still lacking. In the context of global warming, freshwater microbial communities at the base of the ecosystem functioning are increasingly expected to be affected by such events, which imply the recurrent loss of their habitat by desiccation for periods extending from weeks to months.

During a temporal survey of aquatic protist diversity that we carried out in a variety of small freshwater bodies (Simon et al., 2015b), two of the systems, one brook and one pond, experienced complete desiccation for short (ca. 1 and 2 months) and long (ca. 5 months) periods. This offers us the possibility to observe if and how the microbial communities recovered. Here, we describe the temporal evolution along a 2-years monthly sampling of the microbial eukaryotic diversity of those systems, as determined by 454-pyrosequencing of amplified 18S rRNA gene fragments, (i) in water before and after drought periods and (ii) in the underlying dry sediments sampled during droughts. Our results show a relatively rapid recovery of average aquatic communities after prolonged desiccation, highlighting an important resilience of the eukaryotic component of freshwater microbial communities.

## Materials and Methods

### Study Sites and Sampling

Samples were collected monthly, from April 2011 to April 2013, in a pond (La Claye) and a brook (Ru Sainte Anne). These two semi-permanent small freshwater systems are located in the Natural Regional Park of the Haute Vallée de Chevreuse (France, South of Paris). See Simon et al. (2015a) for more details on these sites and the sampling procedure. Briefly, surface water was systematically collected in the morning at ca. 10 a.m. using sterile bottles and processed immediately back in the laboratory (around 25 km away from the sampling site). The whole sampling processing, including nutrient measurements was completed within 4–5 h. Planktonic cells were collected onto 0.2 μm pore-size Nuclepore filters (Whatman) after a pre-filtration step through 5 μm pore-size Nuclepore membranes (Whatman). Filters were then stored frozen at -20°C until DNA extraction. During drought periods, when the freshwater systems underwent complete desiccation, samples were collected by directly scratching the surface of the dry sediment (top 1 cm) with 50 ml sterile Falcon tubes (Becton Dickinson, Biosciences), then transferred to 5 ml sterile cryotubes back to the laboratory and immediately frozen until DNA extraction.

### DNA Extraction, Amplification, and Sequencing of 18S rDNA Fragments

DNA was extracted from 0.25 g of sediment or from cells harvested onto 0.2 μm-pore diameter filters using the PowerSoil DNA extraction kit (MoBio) according to the manufacturer’s instructions (Simon et al., 2015b). DNA was eluted in 80 μl of elution buffer (Tris 10 mM). 18S rDNA fragments including the V4 hypervariable region were amplified using primers EK-565F (5′-GCAGTTAAAAAGCTCGTAGT-3′; Simon et al., 2015a) and 18S-EUK-1134-R-UNonMet (5′-TTTAAGTTTCAGCCTTGCG-3′) biased against Metazoa (Bower et al., 2004). Primers were tagged with different Molecular IDentifiers (MIDs) to allow multiplexing and later differentiation of PCR products from the nine sediment samples along with amplicons from aquatic samples presented in a previous study (Simon et al., 2015b). PCR amplifications were conducted in a total reaction volume of 25 μl, using 1.5 mM MgCl_2,_ 0.2 mM of each dNTP (PCR Nucleotide Mix, Promega), 0.3 μM of each primer, 1–2.5 μl of DNA and 0.5 U HotStart Taq polymerase (Taq Platinum, Invitrogen). Amplification was carried out during 25 cycles (94°C for 30 s, 58°C for 45 s, and 72°C for 90 s), after 3 min of denaturation at 94°C and before a final extension step at 72°C for 10 min. Amplicons from 5 to 8 independent PCR amplifications for each sample were pooled together. Each pool was then purified using the QIAquick PCR purification kit (QIAgen), according to the manufacturer’s instructions. Finally, the same amount of purified PCR products from sediment samples were pooled along with amplicons from plankton samples and pyrosequenced using the 454 GS-FLX Titanium technology from Roche (Beckman Coulter Genomics). Two distinct sets of 0.25 g sediment samples from La Claye collected in December 2011 were treated independently (replicates) from DNA extraction to sequence analysis to assess the reproducibility of our method for sediment samples. We thus processed a total of nine samples: five from La Claye collected from the end of July to December 2011 plus one replicate from the latter sampling month, one from La Claye in September 2012 and two from Ru Sainte Anne in August and September 2012.

### 454 Pyrosequence Analysis

A total of 48,429 pyrosequences were obtained from the nine dry sediment samples. We applied a series of filters to keep only high-quality reads. Sequences with errors in the primer region and/or positions with undetermined bases were eliminated using a local pipeline (Bachy et al., 2013; Simon et al., 2015b). Quality-checked pyrosequences were analyzed with AmpliconNoise (Quince et al., 2011) integrated to our local pipeline in order to eliminate PCR and 454 sequencing errors. After filtering, 34,821 high-quality reads obtained from sediment samples were retained. They were considered together with high-quality reads obtained from aquatic samples of the same systems but between drought periods (Simon et al., 2015b) to build operational taxonomic units (OTUs) using AmpliconNoise (**Table 1**). OTUs were composed of clustered filtered reads, with a 98% identity similarity cut-off. For cautionary reasons, OTUs containing only one sequence were eliminated from the analysis. The most abundant read of each OTU was queried against the PR2 database (Guillou et al., 2013) using BLASTN (Guillou et al., 2013) for taxonomic assignation of the OTUs. Sequences in all OTUs were then attributed to their sample according to their MIDs. Chimerical OTUs were eliminated during a stringent procedure combining both manual and automated steps (Simon et al., 2015a). OTUs affiliated to cryptophyte nucleomorphs were not included in the analysis. Sequences have been deposited at NCBI under the Bioproject number PRNJA305896.

**Table 1:** Total number of reads, OTUs and average diversity estimates for aquatic samples and dry sediment samples collected during droughts.

		No. months	No. reads	No. OTUs	Richness	Simpson index	Evenness
Ru Sainte Anne	Water	22	174297	1956	166.1	0.90	0.68
	Sediment	2	7391	432	220.2	0.97	0.82
	All	24	181688	2141	n.d.	n.d.	n.d.

La Claye	Water	18	187663	1063	83.9	0.78	0.54
	Sediment^∗^	6	23871	913	213.7	0.97	0.81
	All	24	211534	1685	n.d.	n.d.	n.d.

Total	Water	16	361960	2560	129.1	0.84	0.62
	Sediment^∗^	8	31262	1176	215.3	0.97	0.81
	All	24	393222	3132	n.d.	n.d.	n.d.

*n.d., not determined.*

*^∗^Additionally, 3,559 high-quality reads corresponding to one replicate sample from La Claye dry sediment (34,821 sediment reads in total) were analyzed to validate reproducibility, but these data were excluded from this comparative table*.

### Statistical Analysis

Statistical analysis were all conducted using the R software (R Development Core Team, 2013). Diversity and richness estimates were computed based on raw counts of reads attributed to each OTU, using the R package ‘Vegan.’ Richness was estimated by rarefaction, as the expected number of OTUs in a random subsample of each sequence library, having the size of the smallest library (Hurlbert, 1971). Diversity was estimated by the Simpson index, calculated as 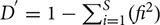 (Simpson, 1949) and evenness as 
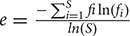
 (Pielou, 1966) with *S* being the observed number of OTUs and *fi* the frequency of each OTUi in the sample. To evaluate overall differences between eukaryotic assemblages, pairwise Bray–Curtis dissimilarities were calculated between all samples (
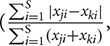
 with x_ji_ and x_ki_ the abundances of OTU_i_ in samples *j* and *k*, respectively, and *S* the number of OTUs observed in libraries *j* and *k*), based on OTU percentages of reads (instead of raw counts to not consider differences due to different numbers of reads). They were computed using the R ‘Vegan’ package (Oksanen et al., 2013). The same package was used to draw Non-metric MultiDimensional Scaling (NMDS) plots comparing communities from the 2-years survey of both ecosystems or on La Claye and Ru Sainte Anne separately. They were based on Bray–Curtis dissimilarities calculated after square-root transformation and Wisconsin standardization (Bray and Curtis, 1957) of OTU percentages. Ellipses were drawn on NMDS plots using the R package ‘Ade4’ (Dray and Dufour, 2007) to highlight whether the communities were collected in water or sediment. Boxplots were drawn with notches to indicate whether the medians of the represented distributions could be considered as different (Chambers et al., 1983).

## Results and Discussion

**FIGURE 1:**
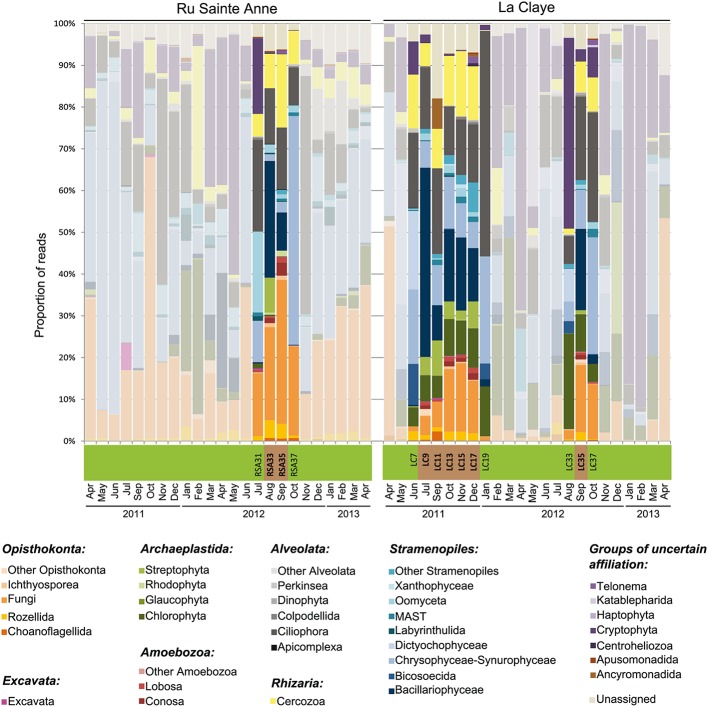
**Phylogenetic composition of microbial eukaryotic communities over a 2-years monthly temporal survey in the brook (Ru) Sainte Anne and the shallow lake La Claye.** Light-colored bars correspond to plankton samples collected over the 2 years (Simon et al., 2015b). Un-masked, bright color bars show the relative proportion of eukaryotic lineages identified in dry sediment during desiccation events as well as in water samples collected in neighboring months right before and after droughts. The horizontal bar below the histograms indicates whether the corresponding histogram bars correspond to water (green) or sediment (brown) eukaryotic communities.

During a previous study on the diversity and seasonal dynamics of microbial eukaryotes of several freshwater systems located at the Natural Park of the Haute Vallée de Chevreuse (France) based on massive 18S rDNA amplicon 454 pyrosequencing (Simon et al., 2015b), two of these systems, a small brook (Ru Sainte Anne) and a peat bog-like substrate pond (La Claye; see Simon et al., 2015a for specific details) experienced droughts of various durations (1–5 months; **Figure 1**). The Ru Sainte Anne dried during August and September 2012. La Claye desiccated from the end of July 2011 to the beginning of December 2011 and during September 2012. We monitored the protist diversity at the top layer of the dry bed sediment (called ‘sediment’ hereafter) using the same approach as for the aquatic communities and compared it to the protist community in aquatic communities before and after drought-induced desiccation events. As sediment communities could be more heterogeneous than aquatic ones, we compared two replicate samples collected independently in La Claye in December 2011. The community compositions detected in both replicates were very similar, as revealed by a Bray–Curtis dissimilarity of only 0.33 (see below), which was far lower than the mean pairwise dissimilarity between all pairs of sediment communities from La Claye (0.68). OTUs defined at 98% similarity threshold on high-quality sequences were used as conservative proxies for microbial eukaryotic species. Over the 2-years monthly survey, a total of 3,132 OTUs were collectively detected in La Claye and the Ru Sainte Anne. More than one third of them (1,176 OTUs) were recorded in the eight sediment samples while 2,560 OTUs were retrieved from the 40 water samples (**Table 1**).

### Global Phylogenetic Composition of Protist Communities from Dry Bed Sediment

Operational taxonomic units from dry sediment and water were attributed to taxonomic groups based on sequence similarity. They distributed among all recognized eukaryotic supergroups [Stramenopile-Alveolata-Rhizaria (SAR), Archaeplastida, Excavata, Amoebozoa, Opisthokonta (Adl et al., 2012)] as well as in several groups of uncertain phylogenetic position, occasionally in very different proportions (**Figure 1**).

**FIGURE 2:**
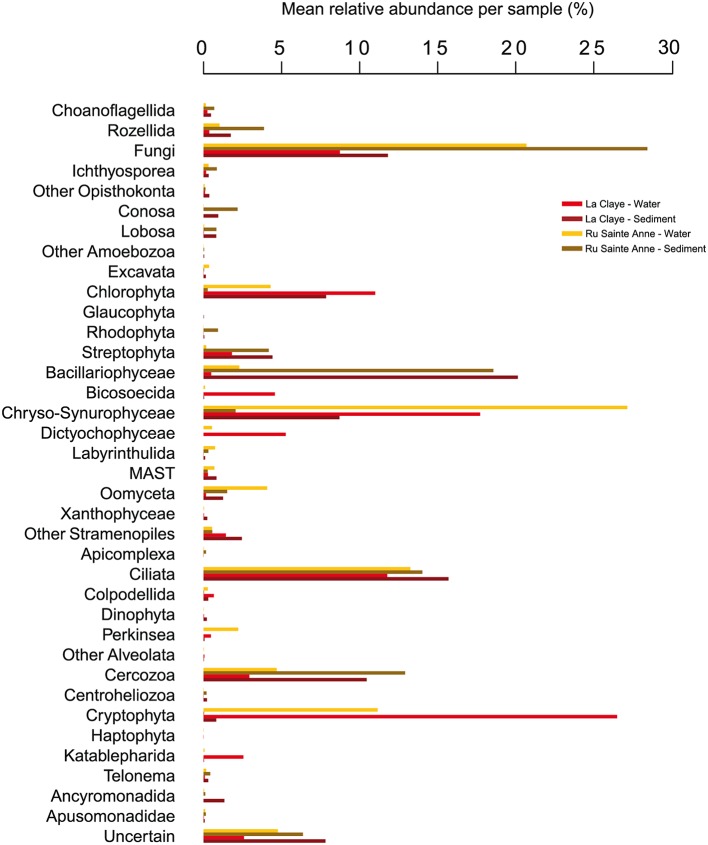
**Average distribution of microbial eukaryotic taxa in water samples and dry sediment during droughts in Ru Sainte Anne and La Claye**.

Stramenopiles constituted the most abundant supergroup in sediment samples from La Claye (33.7% of the reads per sample on average), and the second most abundant in the two samples from the brook (23.3% of the reads per sample on average; **Figures 1** and **2**). This high relative abundance is in accordance with previous records of this supergroup in a wide diversity of environments, from marine and freshwater systems (Richards et al., 2005; Lefèvre et al., 2008; Triadó-Margarit and Casamayor, 2012; Kirkham et al., 2013; Simon et al., 2015a) to soils (e.g., Moon-van der Staay et al., 2006). Members of the Chrysophyceae-Synurophyceae and especially Bacillariophyceae were the most abundant stramenopiles in sediment assemblages, although OTUs affiliated to Labyrinthulida, MAST groups, Oomyceta and Xanthophyceae were also detected. In Ru Sainte Anne, Opisthokonta constituted the dominant supergroup, with 33.9% of reads per sample (average from the two samples) affiliated to that supergroup. They were mostly composed of fungi (28.4% of reads per samples on average), which are an important component of aquatic, sediment and soil habitats (Moon-van der Staay et al., 2006; Lefèvre et al., 2008; Taib et al., 2013). OTUs affiliated to Rozellida-Cryptomycota were moderately abundant (3.9% of reads per sample on average), and Ichthyosporea and Choanoflagellata were also detected. Opisthokonts were less abundant in sediment from La Claye (14.7% of reads per sample on average), but still constituted the third most abundant group in that system. Interestingly, a larger abundance of opisthokonts in the brook as compared to the pond was also detectable in aquatic communities (**Figures 1** and **2**). Alveolates are also common members of freshwater lakes and ponds (Šlapeta et al., 2005; Taib et al., 2013; Simon et al., 2015a) and were always highly abundant in sediment samples from both the brook and the pond (**Figure 1**). In both cases, they were mainly represented by ciliates (**Figures 1** and **2**), which are known for their high abundance and widespread distribution in soils (Foissner, 1997), freshwater (e.g., Sonntag et al., 2006; Lefèvre et al., 2008; Charvet et al., 2012; Mangot et al., 2012) or sediments (e.g., López-García et al., 2003). OTUs affiliated to Archaeplastida (mostly Streptophyta and Chlorophyta) were also abundant in all sediment samples from La Claye and in August 2012 in Ru Sainte Anne (**Figure 1**). Rhizaria, exclusively represented by OTUs affiliated to Cercozoa, were abundant in all sediment samples (**Figure 1**), representing on average 10.4 and 12.9% of reads per sample from La Claye and Ru Sainte Anne, respectively. Amoebozoans represented 1.8 and 3.0% of reads per sample from La Claye and Ru Sainte Anne on average, respectively. Apusozoans, a deep-branching group related to Opisthokonts, were generally detected in low frequency, but reached 7.3% of reads from the sediment community sampled in La Claye in September 2011. In addition, OTUs affiliated to excavates, cryptophytes, katablepharids (only in La Claye), telonemids and centrohelids were also detected in sediment samples, though in relatively low abundances. In summary, eukaryote assemblages from sediment of the dry sediment bed of the studied freshwater systems were highly diverse, belonging to a wide variety of phylogenetic lineages virtually covering the full spectrum of eukaryotic phyla.

### Distinct Communities in Bed Sediment and Water

**FIGURE 3:**
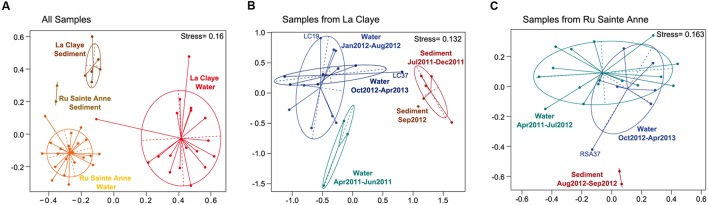
**Non-Metric MultiDimentional Scaling (NMDS) plots corresponding to eukaryote communities from water and dry sediment samples collected along the 2-years survey in Ru Sainte Anne and La Claye (A), La Claye only (B), and Ru Sainte Anne only (C)**. In **(B,C)** ellipses group eukaryote communities of the same periods defined by drought events.

Although, in principle sediment communities should be composed of resident organisms and aquatic cells that sedimented and/or encysted under desiccation conditions, communities detected in the dry sediment during the droughts always differed from communities found in the water column (**Figure 3**). This suggests that aquatic cells entering dormancy at this time composed a tiny fraction of the resident sediment community (or at least the resident community plus a larger, inactive “seed bank” accumulated in the sediment over time). Alternatively, or simultaneously, those dormant cells could be more refractory to lysis, yielding negligible amounts of DNA. Sediment and aquatic communities were nevertheless less different in Ru Sainte Anne than in La Claye, possibly due to the very low depth of the brook (<20 cm) that may favor sediment resuspension.

**FIGURE 4:**
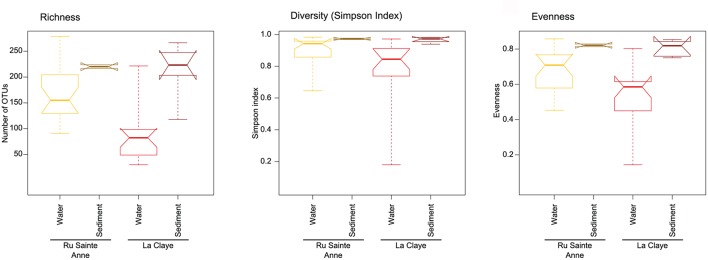
**Differences in richness, diversity and evenness between all water and sediment communities in Ru Sainte Anne and La Claye.** The thick line represents the median of the distribution; the lower and upper limits of the boxes correspond to the first and third quartile respectively. Whiskers extend to the minimal and maximal values. Notches are drawn to indicate whether medians from distinct distributions can be considered as different.

In each of the systems, sediment communities were richer and more diverse (richness, Simpson, and evenness indices) than the planktonic communities. This was visible when comparing all the sediment to all the freshwater samples (**Figure 4**) or when we compared only subsets of samples (4 months) from sediment and aquatic communities collected in La Claye at the same period of the year, 1 year apart (Supplementary Figure S1). Also, eukaryote communities were more similar in the sediment than in the water (**Figure 3**); the mean pairwise Bray–Curtis dissimilarities between communities from the same ecosystem and the same sample type, being of 0.85 and 0.68, respectively (Supplementary Figure S2). The same pattern was obtained when comparing the same number of water and sediment samples from La Claye but also for the same period of the year; the mean pairwise dissimilarity values being of 0.85 and 0.65 respectively (equivalent analyses were not possible for the Ru Sainte Anne due to the restricted number of samples from dry sediment). The higher variability in these indices observed for the planktonic communities could be possibly due to a stronger temporal dynamics as compared with the sediment counterparts (having potentially less buffering capacity) rather than to the larger sampling period. In addition, a comparison of samples from the two systems revealed that, regardless the ecosystem, protist communities were more similar in sediment samples than in water, based on pairwise Bray–Curtis dissimilarities (**Figure 3A** and Supplementary Figure S3). This could be explained by the occurrence of more similar environmental conditions in terms of soil/sediment physico-chemistry as compared to the water samples.

A larger diversity of OTUs in sediment may be explained by an intrinsically higher heterogeneity and substrate complexity of this biotope, but also to the additional presence of resting stages, dead cells or free DNA coming from planktonic communities that sedimented before or during the drought period (despite the fact that few OTUs were detected in both sediment and water samples). It might be thought that differences in taxonomic composition of the eukaryote assemblages between sediment and aquatic habitat could be related to the size-fractionation step applied to retain cells of 0.2–5 μm in size, while no fractionation was applied to sediment communities. However, we previously showed that aquatic protist communities in these systems were highly similar in the 0.2–5 and 5–30 μm size-fractions (Simon et al., 2015a).

The differences observed between aquatic and sediment microbial eukaryote assemblages were also reflected in their overall taxonomic composition. Thus, whereas Bacillariophyta were clearly the dominant stramenopile group in the sediments (19.7% of the reads per sample, on average), they were much less abundant in water (1.5% of the reads per sample, on average; **Figure 2** and Supplementary Figure S4). Similarly, members of several other groups, including the Cercozoa, Amoebozoa and the Rozellida-Cryptomycota were more abundant and diverse in sediment than in water (**Figure 2**). Conversely, some groups, notably the Chrysophyta were clearly dominant in the water samples (26.5 and 11.1% of reads in La Claye and Ru Sainte Anne, on average), but were detected only in very low abundances in sediments (0.03 and 0.8%, respectively). Cryptophyta are known as frequent and quantitatively important members of planktonic communities in freshwaters (Lefranc et al., 2005; Šlapeta et al., 2005; Taib et al., 2013).

Sediment samples were collected during droughts, in summer and autumn only, while planktonic samples covered the four seasons. Yet, the main differences observed here between sediment and aquatic communities at the level of higher taxa, but also at the level of OTUs, are not due to seasonal changes, as all water samples from different seasons cluster together and away from sediment samples (**Figure 3**). Likewise, water and sediment samples collected during the same season (and not at different periods of the year), are as different between them as sediment and water samples collected at different periods of the year.

**FIGURE 5:**
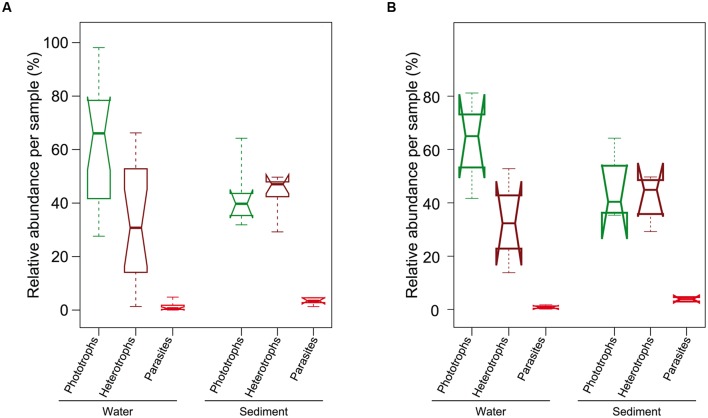
**Distribution of the relative abundance of putative phototrophs, free living heterotrophs and parasites in water and sediment samples from La Claye. (A)** All samples from La Claye; **(B)** four sediment samples from summer and autumn 2011 and four water samples from the same period in 2012. Thick lines indicate median values. The top and below limits of boxes indicate the third and first quartile respectively. Whiskers extend to the minimal and maximal values. Notches are drawn to indicate whether medians from distinct distributions can be considered as different. Putative phototrophs, free-living heterotrophs and parasites were defined as previously (Simon et al., 2015b).

Differences in taxonomic community composition were concomitant to differences in the functional composition of the eukaryote communities in La Claye. Phototrophic organisms generally dominated planktonic communities while putative heterotrophic eukaryotes seemed to slightly dominate in the sediment communities in La Claye pond (**Figure 5**). In addition, putative parasites, especially rozellids, were more abundant in the sediment than in the aquatic habitat. These results could underscore the higher importance of organic matter (inert and alive) in the sediment that may favor predators and saprophages. Because there were only two sediment samples of the same period for Sainte Anne Brook, the analysis could not be reliably done for this ecosystem.

### Recovery of Aquatic Microbial Eukaryotic Communities After Droughts

A drought leading to the total drying up of small freshwater ecosystems constitutes an important stress that entails the temporary disappearance of the normal biotope for planktonic members of the community. This, in principle, could trigger drastic changes in the aquatic communities after water refill if members of the aquatic community are not able to produce resting stages and/or enter dormancy, and if the desiccation period lasts for long and those dormant stages have differential resistance to desiccation and other physico-chemical stresses derived from it. The community structure might not recover from such disturbance, which, in turn, might have consequences affecting ecosystem functioning. However, in this study, we show two remarkable examples of community recovery after such extreme drought events. We did not observe any significant aquatic community composition shift neither after droughts of 1–2 months in both La Claye pond and Ru Sainte Anne nor after a desiccation period of ca. 5 months in La Claye, from late July 2011 to early January 2012 (**Figure 3**). In the latter case, the protist community that was observed just after La Claye pond was filled up again in January 2012 (LC19) was typical of aquatic eukaryotes, while it was slightly closer to the sediment community after the shorter second drought in summer 2012 (LC37; **Figure 3B**). A similar case could be made for Ru Sainte Anne, the RSA37 sample collected after the drought period was somewhat intermediate between typical sediment and water samples (**Figure 3C**). However, aquatic communities sampled 1 month later (November 2012, samples LC39 and RSA39, respectively) had again a typical signature of an aquatic community (**Figure 3**). This difference could simply reflect how close to the sampling the rain episodes that refilled the systems were. The two major rain episodes after the first drought occurred 12-7 and 29-22 days before sampling LC19 (January 2012) while they took place 19-13 and 7-1 days before sampling LC37 and RSA37 (October 2012) for the second drought (Supplementary Figure S5).

The recurrent recovery in communities such as that of La Claye, even after extended periods of drought, indicates a strong resilience of aquatic protists. The recovery takes place rapidly, within a month. Although such a rapid recovery was not observed for sediment bacterial assemblages in a semi-permanent stream subjected to drought (Rees et al., 2006), examples of fast recovery of planktonic microbial communities subjected to drastic water column mixing exist (Shade et al., 2012b). Even if additional observations in other systems will be required to check how general the resilience of these microbial communities is, our results suggest that aquatic communities in small shallow ecosystems from temperate areas are well-adapted to stressful conditions, such as desiccation, for extended periods of time.

Small freshwater ecosystems are highly dynamic and their aquatic communities are exposed to important and rapid seasonal variations derived from changes in environmental conditions (Bamforth, 1958; Simon et al., 2015b). Short and long droughts can occur regularly and seasonally in small freshwater systems. Macro-organism assemblages undergoing regular seasonal drought are characterized by a strong recovery capacity, most likely because organisms that use refuge or can adopt a life-stage that can stand desiccation are selected (Lake, 2003). Likewise, microbial communities inhabiting particular environments such as ponds and brooks are composed by taxa capable of generating resting forms that resist desiccation. Indeed, we identified a few OTUs that appeared to be very resistant to drought events in our two systems. For instance, OTU 290 (93% identical to DQ104595, *Rozella* sp.) and OTU 356 (99% identical to DQ244023, *Stichotrichia*) were detected during the whole 2-years survey, including sediment and planktonic assemblages, in Ru Sainte Anne and La Claye pond, respectively.

The planktonic eukaryote community in Ru Sainte Anne and La Claye pond may thus be partly composed of taxa capable of forming resting stages that can develop again when the aquatic habitat is restored. In addition to dormancy, immigration from geographically close and not so drastically disturbed ecosystems might be another potential explanation for microbial recovery (Shade et al., 2012a), since those systems can serve as reservoirs of organisms that are not able to survive in dry sediment conditions. However, microorganisms lacking resting forms and sensitive to drought are precisely the less likely to survive migration, during which desiccation and exposure to ultraviolet radiation can occur, especially among individualized water bodies such as ponds. In addition, the microbial communities found in different freshwater systems, even if they are very closely situated, can be very different depending on the local physico-chemical conditions (Simon et al., 2015b), which limits their role as reservoirs of active organisms for subsequent dispersal. All this suggests that the capacity to enter dormancy via the generation of resting forms (e.g., cysts, spores or modified, resistant metabolic stages) is the major explanation for the resilience of microbial communities observed in shallow freshwater ecosystems periodically undergoing droughts. Further studies aimed to distinguish active from dormant microorganisms in sediments (e.g., via rRNA versus rDNA comparison) would help testing this hypothesis.

## Author Contributions

PL-G, LJ, and DM conceived the research; MS, LJ, PB, DM, and PL-G did the sampling; MS and PB carried out the experimental analyses; MS, PD, and DM analyzed the sequences; MS and GR did the statistical analyses; MS, LJ, and PL-G wrote the manuscript.

## Conflict of Interest Statement

The authors declare that the research was conducted in the absence of any commercial or financial relationships that could be construed as a potential conflict of interest.
