# Supplementary material for: Resilience of Freshwater Communities of Small Microbial Eukaryotes Undergoing Severe Drought Events
Source: Front Microbiol. 2016 May 30;7:812. doi: 10.3389/fmicb.2016.00812 (PMC4885337; doi:10.3389/fmicb.2016.00812)
Supplement: Supplementary file 1 [file Presentation_1.PDF]

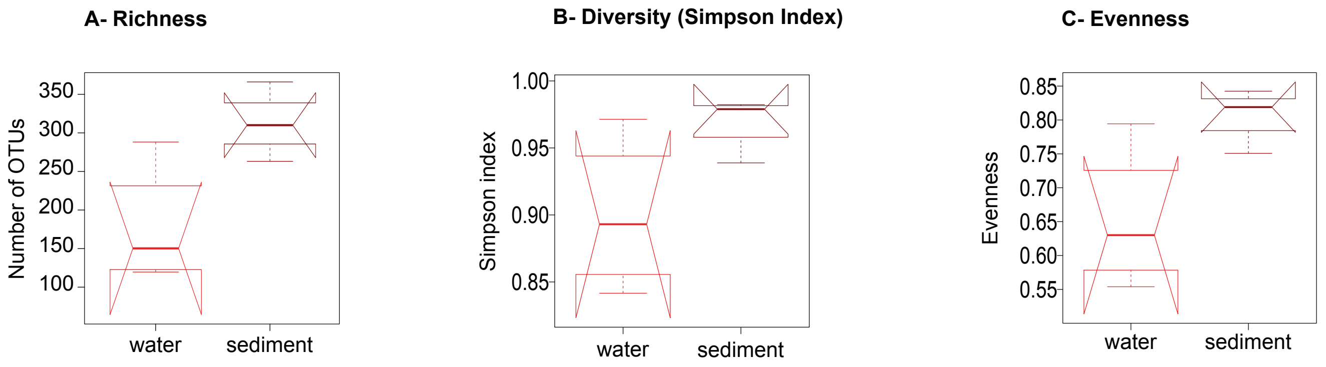

Figure S1. Distributions of richness and diversity indices among four sediment samples from summer and autumn 2011 and four water samples from the same period in 2012 in La Claye. Richness is defined as the expected number of OTUs in a subsample of the same size as the smallest sample included in the study. The thick line represents the median of the distribution, the lower and upper limits of the boxes correspond to the first and third quartile respectively. Whiskers extend to the minimal and maximal values. Notches are drawn to indicate whether medians from distinct distributions can be considered as different.

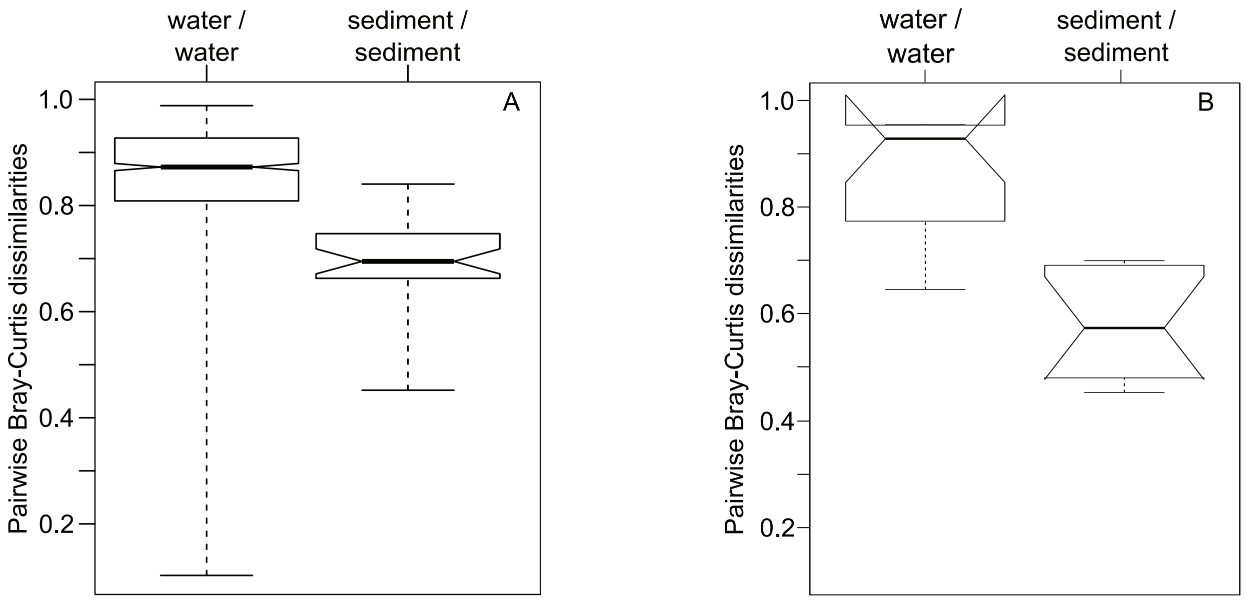

Figure S2. Distribution of pairwise Bray-Curtis dissimilarities conducted on OTU percentages, between all (A) samples from the same ecosystem and of the same type (water or sample), or (B) between four sediment samples from summer / autumn 2011 and between four water samples from the same period in 2012 (La Claye). Thick horizontal bars represent the median of distributions, the lower and upper borders of the box correspond to the first and third quartiles respectively. Whiskers extend to the minimal and maximal values. Non overlapping notches are strong evidence that medians differ.

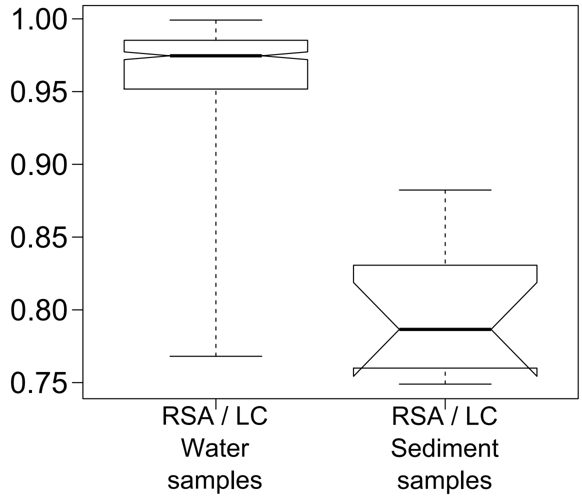

Figure S3. Distribution of pairwise Bray-Curtis dissimilarities between water or sediment samples from distinct ecosystems. RSA, Ru Sainte Anne; LC, La Claye. Thick lines correspond to the medians of distributions. Lower and upper limits of boxes indicate the first and third quartiles, respectively. Whiskers extend to the minimal and maximal values of distributions. Non overlapping notches are a strong evidence that medians differ.

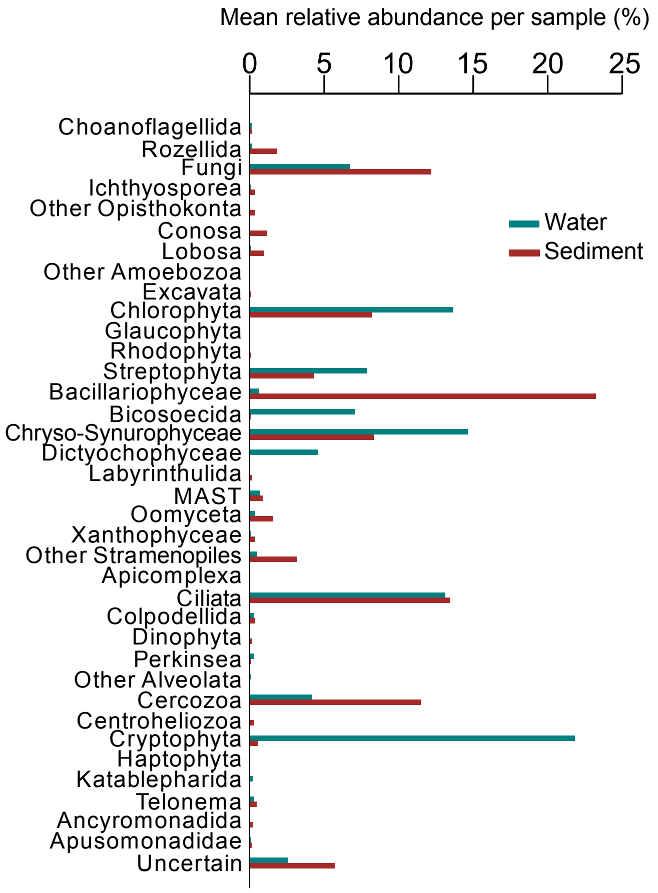

Figure S4. Taxonomic composition of sediment and water communities from La Claye. Mean relative abundance by sample were calculated on four sediment samples from summer and autumn 2011 and four water samples from the same period in 2012.

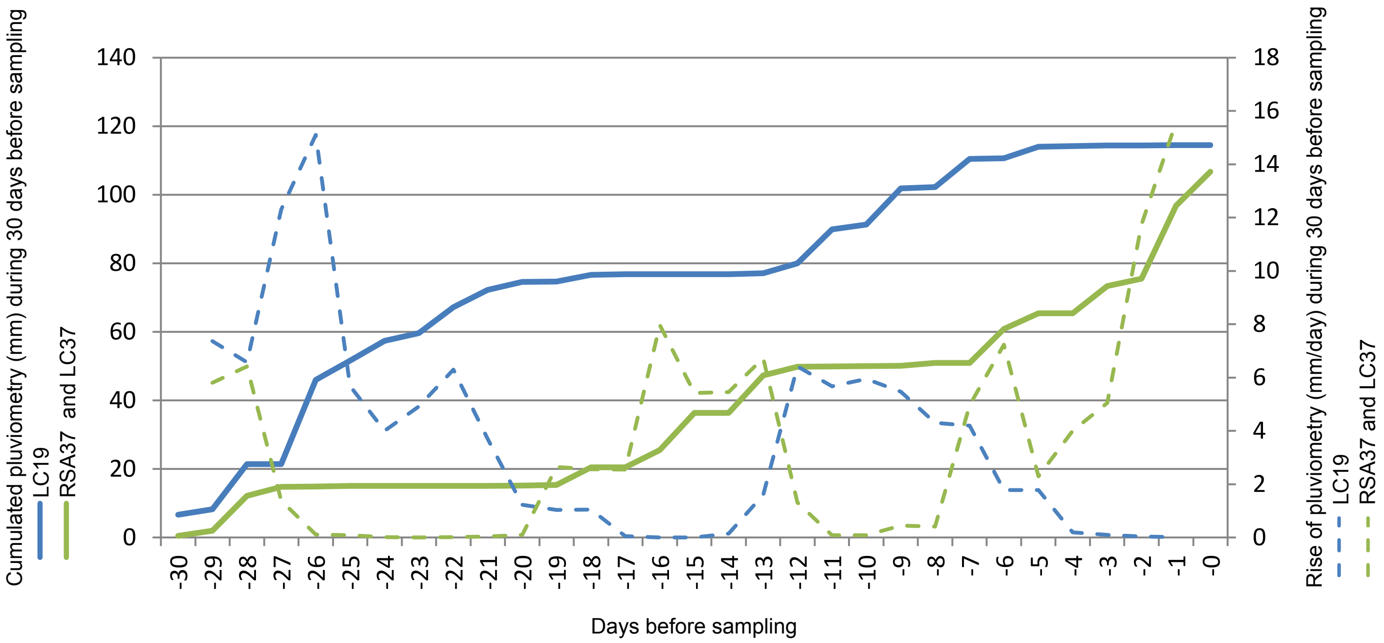

Figure S5. Pluviometry during the month before the first samplings following rewetting. Rise of puviometry at day d is estimated as half the difference between puviometry at day d+1 and at d-1.
